# Semaphorin-3A Promotes Degradation of Fragile X Mental Retardation Protein in Growth Cones *via* the Ubiquitin-Proteasome Pathway

**DOI:** 10.3389/fncir.2020.00005

**Published:** 2020-02-28

**Authors:** Masaru Takabatake, Yoshio Goshima, Yukio Sasaki

**Affiliations:** ^1^Functional Structure Biology Laboratory, Department of Medical Life Science, Yokohama City University Graduate School of Medical Life Science, Yokohama, Japan; ^2^Department of Molecular Pharmacology and Neurobiology, Yokohama City University Graduate School of Medicine, Yokohama, Japan

**Keywords:** semaphorin, fragile X mental retardation protein, fragile X syndrome, axon guidance, microtubule-associated protein 1B, growth cone, ubiquitin, proteasome

## Abstract

Fragile X mental retardation protein (FMRP) is an RNA-binding protein that regulates local translation in dendrites and spines for synaptic plasticity. In axons, FMRP is implicated in axonal extension and axon guidance. We previously demonstrated the involvement of FMRP in growth cone collapse *via* a translation-dependent response to Semaphorin-3A (Sema3A), a repulsive axon guidance factor. In the case of attractive axon guidance factors, RNA-binding proteins such as zipcode binding protein 1 (ZBP1) accumulate towards the stimulated side of growth cones for local translation. However, it remains unclear how Sema3A effects FMRP localization in growth cones. Here, we show that levels of FMRP in growth cones of hippocampal neurons decreased after Sema3A stimulation. This decrease in FMRP was suppressed by the ubiquitin-activating enzyme E1 enzyme inhibitor PYR-41 and proteasome inhibitor MG132, suggesting that the ubiquitin-proteasome pathway is involved in Sema3A-induced FMRP degradation in growth cones. Moreover, the E1 enzyme or proteasome inhibitor suppressed Sema3A-induced increases in microtubule-associated protein 1B (MAP1B) in growth cones, suggesting that the ubiquitin-proteasome pathway promotes local translation of MAP1B, whose translation is mediated by FMRP. These inhibitors also blocked the Sema3A-induced growth cone collapse. Collectively, our results suggest that Sema3A promotes degradation of FMRP in growth cones through the ubiquitin-proteasome pathway, leading to growth cone collapse *via* local translation of MAP1B. These findings reveal a new mechanism of axon guidance regulation: degradation of the translational suppressor FMRP *via* the ubiquitin-proteasome pathway.

## Introduction

The formation of precise neural networks during development is essential for proper neural functions. One of the most important events for building a precise neural network is the wiring of axonal projections towards the correct targets to make proper synapses. Precise axonal projection is a consequence of appropriate axonal navigation in response to environmental cues (Tessier-Lavigne and Goodman, 1996). During axonal navigation, a very motile and unique amoeba-like structure at the axonal terminal, called a growth cone, determines the elongation route by guidance in response to attractive or repulsive cues (Dent et al., 2011; Kolodkin and Tessier-Lavigne, 2011). Attractive cues, such as netrin-1 and brain-derived neurotrophic factor (BDNF), elicit cytoskeletal polymerization and exocytic membrane trafficking at the stimulated side of the growth cone, resulting in an extension of the growth cone area on the stimulated side (Dent et al., 2011; Tojima and Kamiguchi, 2015). In contrast, repulsive guidance cues, such as Semaphorin-3A (Sema3A) and Slit, elicit cytoskeletal depolymerization and endocytic membrane trafficking at the stimulated side of the growth cone and are involved in growth cone collapse on this side. These changes of growth cone morphology (enlargement or collapse) in response to guidance cues lead to the growth cone turning towards or away from guidance cues.

An increasing body of evidence has demonstrated that local translation plays an important role in axon guidance *via* morphological changes of growth cones, followed by growth cone turning (Campbell and Holt, 2001; Wu et al., 2005; Leung et al., 2006; Piper et al., 2006; Yao et al., 2006). For example, netrin-1 or Sema3A induced attractive turning or collapse of growth cones isolated from cell bodies in a protein-synthesis-dependent manner (Campbell and Holt, 2001). Transcriptome analysis of growth cones revealed that mRNAs for cytoskeletal and membrane trafficking proteins are localized (Zivraj et al., 2010). RNA-binding proteins (RBPs) have been recognized to regulate local translation of these mRNAs for growth cone turning and collapse (Hörnberg and Holt, 2013). In the case of attractive cues, zipcode binding protein 1 (ZBP1), an RBP bound to β-actin mRNA, is required for growth cone turning induced by BDNF and netrin-1 *via* local translation of β-actin mRNA (Leung et al., 2006; Yao et al., 2006; Welshhans and Bassell, 2011). β-actin mRNA and ZBP1 localized to the stimulated side of growth cones, indicating that ZBP1 regulates local translation of β-actin on the stimulated side (Leung et al., 2006; Yao et al., 2006). Phosphorylation of ZBP1 plays an important role in regulating local translation of β-actin mRNA and growth cone turning in response to BDNF and netrin-1 (Sasaki et al., 2010; Welshhans and Bassell, 2011). These results suggest that localization of mRNA-binding proteins on the stimulated side and posttranslational modifications, such as phosphorylation, are important to regulate local translation for growth cone turning. With regard to repulsive cues, we demonstrated that fragile X mental retardation protein (FMRP), an mRNA-binding protein encoded by the causative gene of Fragile X syndrome (FXS; Bear et al., 2004; Darnell and Klann, 2013; Richter et al., 2015), is involved in Sema3A-induced growth cone collapse in a protein-synthesis-dependent manner (Li et al., 2009). However, it remains unclear how Sema3A effects FMRP localization in growth cones, or how FMRP regulates local translation and growth cone collapse in response to repulsive cues.

In this study, we investigated changes in the localization of FMRP in growth cones in response to Sema3A. We showed that FMRP levels in growth cones decreased gradually *via* the ubiquitin-proteasome pathway during Sema3A stimulation. Inhibitors of the ubiquitin-proteasome pathway attenuated Sema3A-induced increases in microtubule-associated protein 1B (MAP1B) in growth cones, as a result of FMRP-dependent local translation (Li et al., 2009). These inhibitors also suppressed the Sema3A-induced growth cone collapse. Thus, our findings suggest an important role of FMRP degradation elicited by ubiquitination, one of posttranslational modifications, in Sema3A-induced local translation and growth cone collapse.

## Materials and Methods

### Dissociated Mouse Hippocampal Neuron Culture

**Figure 1:**
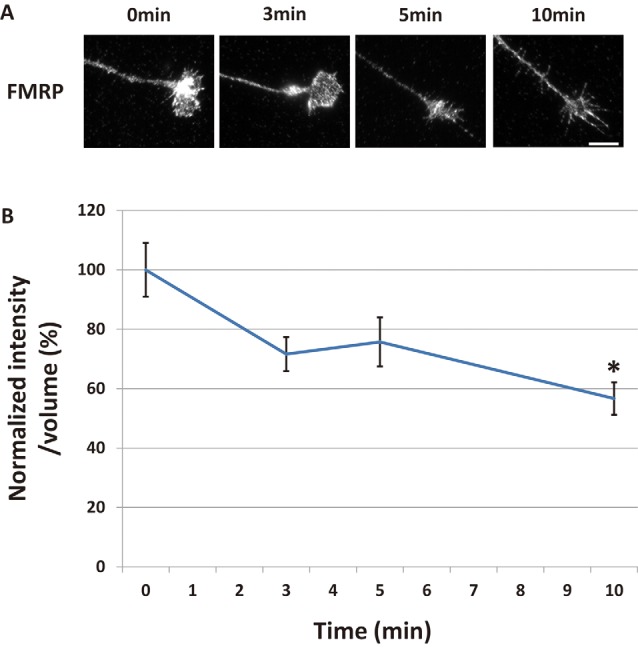
Fragile X mental retardation protein (FMRP) in growth cones decreased time-dependently after Semaphorin-3A (Sema3A) stimulation. **(A)** Immunofluorescence images of anti-FMRP antibody staining in growth cones of dissociated mouse hippocampal neurons. Fluorescence intensities of FMRP in growth cones were gradually reduced by Sema3A stimulation. Scale bar: 10 μm. **(B)** Quantification of fluorescence intensities of FMRP signals in growth cones for time-course experiments as in **(A)**. Data represent mean ± SEM for *n* = 10. *p < 0.05, significantly different from 0 min using one-way analysis of variance (ANOVA) with Tukey’s multiple comparison test.

Hippocampi were removed from mouse E16 embryos and treated with Hank’s Balanced Salt Solution (HBSS) containing 0.25% trypsin (Invitrogen) at 37°C for 5 min. After trypsinization, cells were plated at a density of 3,000 cells/cm^2^ on 15 μg/mL poly-L-lysine (PLL) pre-coated coverslips, and cultured for 3 days in Neurobasal medium containing GlutaMax and B-27 supplement (Invitrogen). Dissociated cultures were used for earlier experiments (Figure 1). Later, neuron ball culture was used to obtain growth cone data more easily than dissociated cultures. Results from dissociated cultures and neuron ball cultures were not significantly different.

### Neuron Ball Culture

Neuron ball cultures were prepared as previously described (Sasaki et al., 2014; Parvin et al., 2019). Since thousands of axons extend radially from neuron ball culture as, like explants of dorsal root ganglia, it is easier to analyze growth cone images. Mouse hippocampi were dissected from E16 embryos. Hanging drops (neuron balls) containing 10,000 cells per drop were maintained for 3 days inside the top covers of 100-mm dishes, which contained water in the bottom dish to maintain humidity. “Neuron balls” were then mechanically placed at 5-mm intervals on PLL-coated dishes containing Neurobasal medium supplemented with GlutaMax and B-27.

### Immunostaining and Growth Cone Collapse Assay

Immunostaining and growth cone collapse assay of neuron ball and dissociated cultures were performed using purified recombinant chick Sema3A, as previously described (Goshima et al., 1995). Neuron ball or dissociated cultures were stimulated with Sema3A for 10 min, except for measurements of the immunofluorescence intensity of ubiquitinated proteins, for which stimulation was performed for 5 min. Cultured neurons were stimulated with 0.3 nM Sema3A for immunostaining and 0.1 nM Sema3A for the collapse assay. After stimulation, neurons were fixed with 4% paraformaldehyde in phosphate-buffered saline at room temperature for 30 min, followed by permeabilization for 5 min using 0.3% Triton X-100 diluted in Tris-buffered saline [TBS; 50 mM Tris-HCl (pH 7.4) and 150 mM NaCl]. In some experiments, the dynein ATPase inhibitor erythro-9-(2-hydroxy-3-nonyl)-adenine (EHNA, 50 μM; R&D Systems), ubiquitin-activating enzyme (E1) inhibitor PYR-41 (1 μM; Sigma Aldrich), or proteasome inhibitor MG132 (15 μM; Sigma Aldrich) was applied for 30 min before stimulation. Prior to staining, fixed samples were blocked for over 1 h in blocking buffer (TBS containing 1.5% normal goat serum). Samples were incubated with anti-FMRP (1C3, 1:1,000; Millipore), anti-ubiquitinated protein (FK2, 1:1,000; Millipore), or anti-MAP1B (AA6, 1:1,000; Abcam) diluted in the antibody diluent Can Get Signal (Toyobo) at 4°C overnight. The following day, samples were incubated at room temperature for over 1 h with Alexa Fluor 555-conjugated secondary antibodies (ThermoFisher), and 5-(4,6-dichlorotriazinyl) aminofluorescein (DTAF; ThermoFisher) to visualize growth cone morphology and normalize the volume of growth cones. For the collapse assay, growth cones were stained with CF488A Phalloidin (1:400; Biotium) overnight. All data for immunofluorescence intensities and collapse rates were analyzed using one-way or two-way analysis of variance (ANOVA) with Tukey’s multiple comparison test.

The effect of EHNA on retrograde transport in axons was visualized by live-cell imaging with LysoTracker Red DND-99 dye (ThermoFisher), which was applied to cortical neuron ball cultures in a glass-bottom dish at a final concentration of 75 nM for 1 h at 37°C. EHNA (final concentration: 50 μM) or vehicle control was applied to the culture for the final 10 min of incubation with the LysoTracker dye. Cultures were washed three times with HBSS in the absence or presence of 50 μM EHNA, and then the medium was changed to phenol red-free Neurobasal medium containing 25 mM HEPES (pH 7.3), B-27, GlutaMax, and 100 mM pyruvate with or without 50 μM EHNA. Lysosomes stained with LysoTracker dye were imaged over 1 min, with image acquisition every 1 s.

### Microscopy and Imaging

Immunofluorescence images were captured with an inverted microscope (Nikon Eclipse Ti-E) equipped with an iXON3 CCD camera (Andor Technology) using a 60× oil immersion lens. Quantitative measurements were performed using ImageJ software (imagej.nih.gov). DTAF fluorescence was measured in the growth cone area and an adjacent area (as background fluorescence), and then the background value was subtracted from the value of growth cone. To normalize immunofluorescence intensities of FMRP, ubiquitinated proteins, and MAP1B in growth cones, ratios between each immunofluorescence intensity and DTAF intensity were calculated for each growth cone.

## Results

### Sema3A Induced Decreased FMRP in Growth Cones

We previously reported the possibility of FMRP having a critical role in Sema3A-induced collapse in hippocampal neurons (Li et al., 2009). To elucidate the role of FMRP in Sema3A-induced growth cone collapse, we investigated changes of FMRP localization in growth cones of hippocampal neurons during Sema3A stimulation. FMRP localized as granules in stems and some filopodia of growth cones (Figure 1A). After Sema3A stimulation at 0.3 nM, FMRP in growth cones gradually decreased for up to 10 min, as detected with an anti-FMRP antibody (Figure 1A). We next measured the fluorescence intensities of FMRP in growth cones at specified times (Figure 1B). To correct for changes in growth cone volume during the collapse, we labeled total proteins of cells using DTAF. To normalize the fluorescence intensity of FMRP, we divided its value by the fluorescence intensity of DTAF of the same growth cone area. Three minutes after Sema3A stimulation, the normalized fluorescence intensity of FMRP in growth cones started to decrease, which became significant at 10 min (Figure 1B). These results indicate that FMRP in growth cones decreased in a time-dependent manner after Sema3A stimulation.

### Decreased FMRP in Growth Cones Was Suppressed by Inhibition of the Ubiquitin-Proteasome Pathway

**Figure 2:**
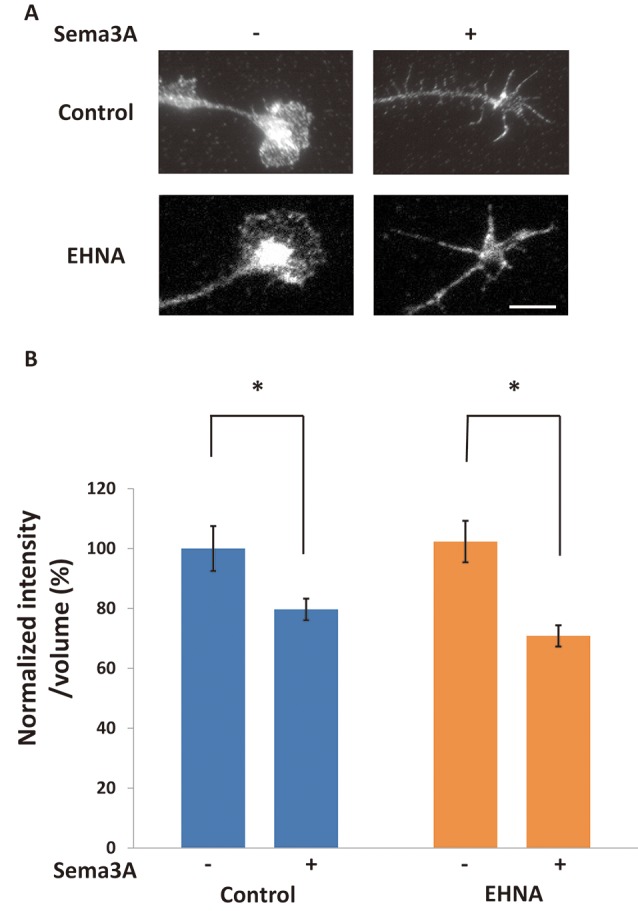
The blockage of retrograde transport did not affect Sema3A-induced decreases in FMRP in growth cones. **(A)** Immunofluorescence images of FMRP in growth cones of neuron ball culture in the presence or absence of EHNA, a dynein inhibitor, with or without Sema3A stimulation. The application of EHNA did not suppress the decrease in FMRP. Scale bar: 10 μm. **(B)** Quantification of fluorescence intensities of FMRP in growth cones as in **(A)**. Data represent mean ± SEM for *n* = 17. *p < 0.05, significantly different from Sema3A (-) using two-way ANOVA with Tukey’s multiple comparison test.

**Figure 3:**
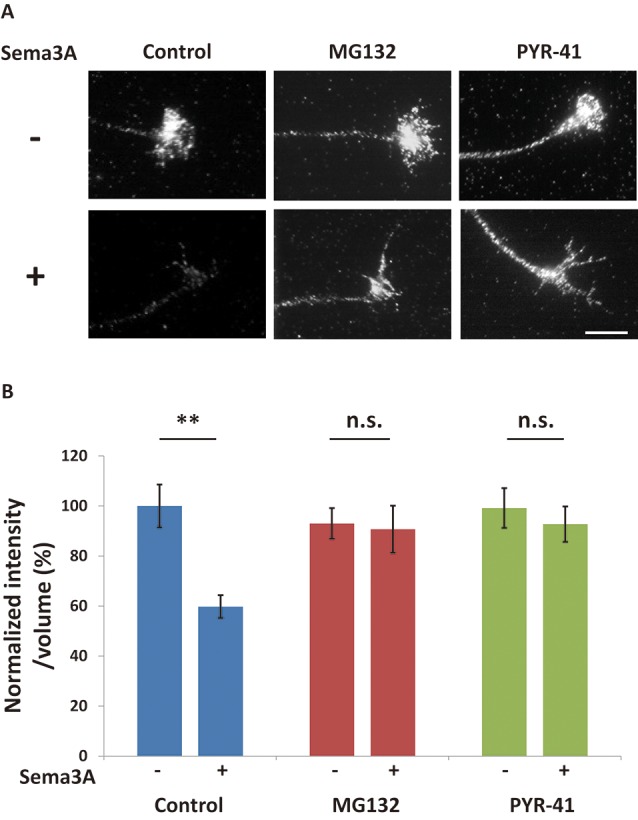
The ubiquitin-proteasome pathway is involved in Sema3A-induced decreases in FMRP in growth cones. **(A)** Immunofluorescence images of FMRP in growth cones in the presence or absence of ubiquitin-proteasome pathway inhibitors. MG132 (a proteasome inhibitor) and PYR-41 (a ubiquitin-activating enzyme inhibitor) suppressed Sema3A-induced decreases in FMRP fluorescence. Scale bar: 10 μm. **(B)** Quantification of fluorescence intensities of FMRP in growth cones as in **(A)**. Data represent mean ± SEM for *n* = 10. **p < 0.01, significantly different from Sema3A (-) using two-way ANOVA with Tukey’s multiple comparison test. n.s., not significant.

There are two possibilities to explain the decrease in FMRP in growth cones after Sema3A stimulation: retrograde transport of FMRP from growth cones and/or degradation of FMRP in growth cones. We previously demonstrated that Sema3A stimulation elicits retrograde transport from axons to cell soma (Li et al., 2004). Thus, we first examined the effect of a dynein ATPase inhibitor, EHNA, on the observed decrease of FMRP in growth cones (Figure 2). In cortical neuron ball cultures, retrograde transport visualized by LysoTracker was observed along the axons in control condition (Supplementary Video S1). However, EHNA treatment inhibited the retrograde transport (Supplementary Video S2). EHNA had no significant effect on Sema3A-induced decreases of FMRP from growth cones (Figure 2B), indicating that localization of FMRP is unaffected by retrograde transport-activated Sema3A signaling. Next, we examined the possibility that Sema3A effects FMRP degradation in growth cones. As FMRP is degraded by the ubiquitin-proteasome pathway in response to 3,5-dihydroxyphenylglycine (DHPG) stimulation in dendrites (Nalavadi et al., 2012), we examined the effects of ubiquitin-proteasome pathway inhibitors MG132 (proteasome inhibitor) and PYR-41 (ubiquitin-activating enzyme E1 enzyme inhibitor) on FMRP localization in growth cones (Figure 3). Fluorescence intensity of FMRP in growth cones decreased significantly when Sema3A was applied without these inhibitors; however, MG132 and PYR-41, unlike EHNA, suppressed the Sema3A-induced decrease in FMRP. Taken together, these results suggest that the observed decreases in FMRP in growth cones in response to Sema3A were mediated by the ubiquitin-proteasome pathway, but not retrograde transport.

### Ubiquitination Was Induced in Growth Cones in Response to Sema3A

**Figure 4:**
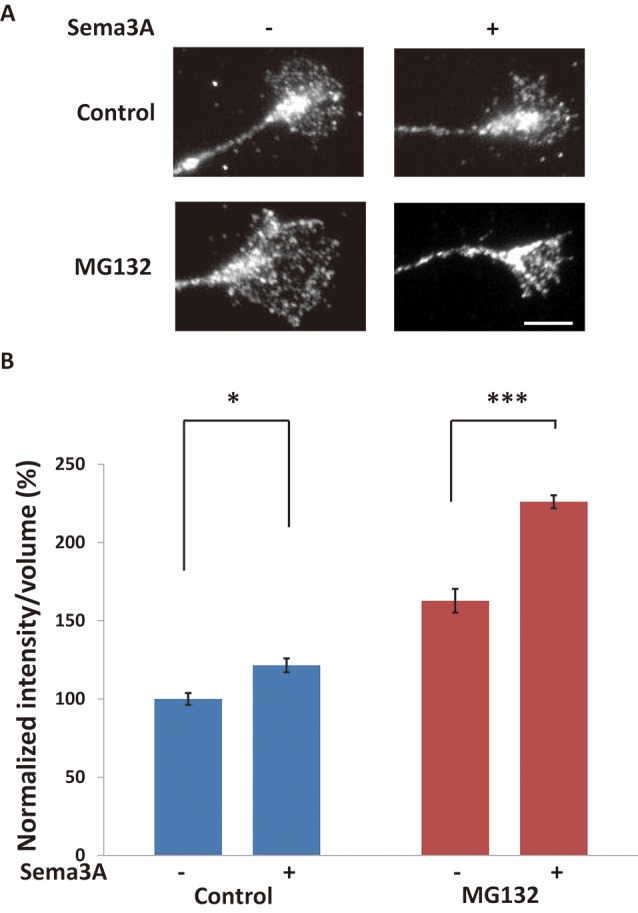
Sema3A induced protein ubiquitination in growth cones. **(A)** Immunofluorescence images of anti-ubiquitinated protein antibody (FK2) staining in growth cones. Sema3A treatment for 5 min increased the fluorescence intensity of ubiquitinated proteins in growth cones. MG132 enhanced the intensities of ubiquitinated proteins in growth cones stimulated by Sema3A more significantly than controls. Scale bar: 10 μm. **(B)** Quantification of fluorescence intensities of ubiquitinated proteins in growth cones as in **(A)**. Data represent mean ± SEM for *n* = 10. *p < 0.05, ***p < 0.001, significantly different from Sema3A (-) using two-way ANOVA with Tukey’s multiple comparison test.

To investigate whether ubiquitination was promoted by the Sema3A signal in growth cones, we examined the ubiquitination of proteins in growth cones using an anti-ubiquitinated protein antibody to recognize mono- and polyubiquitinated protein conjugates (but not free ubiquitin; clone FK2; Figure 4). First, hippocampal neurons were stimulated for 10 min with Sema3A (the same incubation time used for the results shown in Figures 1–3). However, there was no difference in ubiquitinated protein intensity in growth cones with or without Sema3A (data not shown). We considered the possibility that once ubiquitinated, proteins such as FMRP were degraded by the proteasome during the 10-min incubation with Sema3A. Thus, we shortened the incubation time with Sema3A to 5 min. As a result, the intensity of ubiquitinated proteins was significantly increased in growth cones (4B). Moreover, the proteasome inhibitor MG132 enhanced the observed increase in ubiquitinated proteins. These results demonstrate that the Sema3A signal promoted the ubiquitination of proteins in growth cones.

### Involvement of the Ubiquitin-Proteasome Pathway in Sema3A-Induced Increase of MAP1B in Growth Cones and Growth Cone Collapse

**Figure 5:**
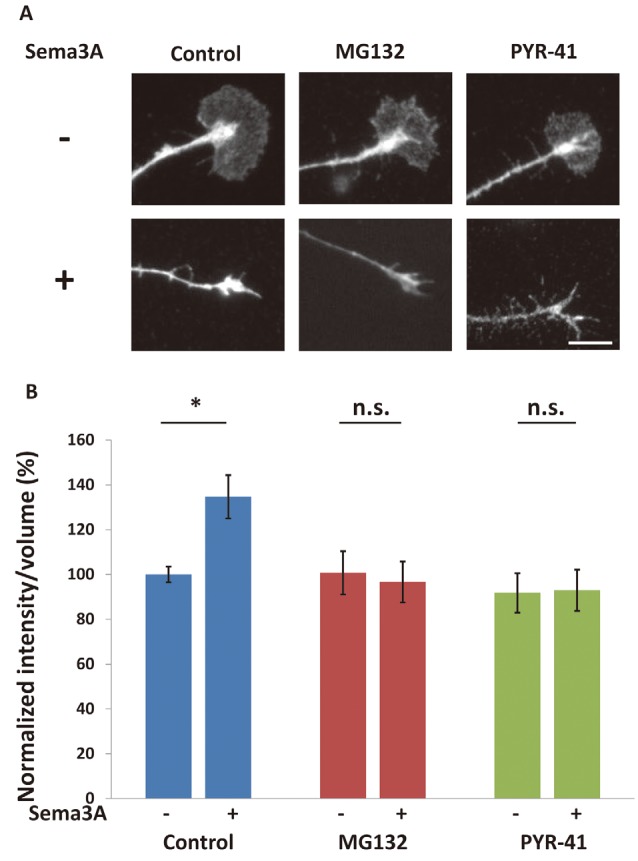
Sema3A-induced increase in microtubule-associated protein 1B (MAP1B) in growth cones was suppressed by ubiquitin-proteasome pathway inhibitors. **(A)** Immunofluorescence images of anti-MAP1B antibody staining in growth cones. MG132 and PYR-41 suppressed Sema3A-induced increases in the intensity of MAP1B in growth cones. Scale bar: 10 μm. **(B)** Quantification of fluorescence intensities of MAP1B in growth cones as in **(A)**. Data represent mean ± SEM for *n* = 10. *p < 0.05, significantly different from Sema3A (-) using two-way ANOVA with Tukey’s multiple comparison test. n.s., not significant.

We previously reported that Sema3A induced an increase of MAP1B expression in distal axons and growth cones in a translation-dependent manner (Li et al., 2009). As such increases in MAP1B, whose mRNA is a target of FMRP, were suppressed in *Fmr1*-KO mice, Sema3A is thought to promote local translation of MAP1B in axons and growth cones in a manner mediated by FMRP (Li et al., 2009). Thus, we investigated the role of the ubiquitin-proteasome system in the local translation of MAP1B. Sema3A induced increased MAP1B intensities in the stems of growth cones (Figure 5). The ubiquitin-proteasome pathway inhibitors PYR-41 and MG132 suppressed this increase in MAP1B immunofluorescence after Sema3A stimulation (Figure 5), indicating that the ubiquitin-proteasome pathway was involved in Sema3A-induced increases in MAP1B in growth cones. Collectively, these results suggest that local translation of MAP1B in growth cones in response to Sema3A was promoted by the ubiquitin-proteasome pathway.

**Figure 6:**
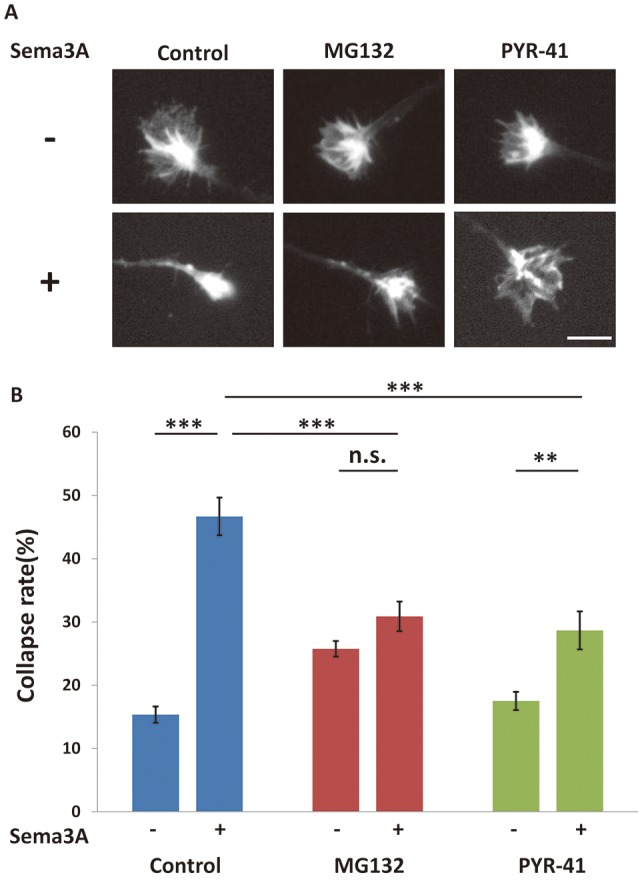
The Sema3A-induced collapse was suppressed by ubiquitin-proteasome pathway inhibitors. **(A)** Immunofluorescence images of phalloidin staining in growth cones. The Sema3A-induced collapse was suppressed in the presence of ubiquitin-proteasome pathway inhibitors (MG132 and PYR-41), Scale bar: 10 μm. **(B)** Quantification of collapse rates of growth cones as in **(A)**. In the presence of ubiquitin-proteasome pathway inhibitors, Sema3A-induced collapse was partially suppressed. Data represent mean ± SEM for *n* = 15. **p < 0.01, ***p < 0.001, significantly different using two-way ANOVA with Tukey’s multiple comparison test. n.s., not significant.

**Figure 7:**
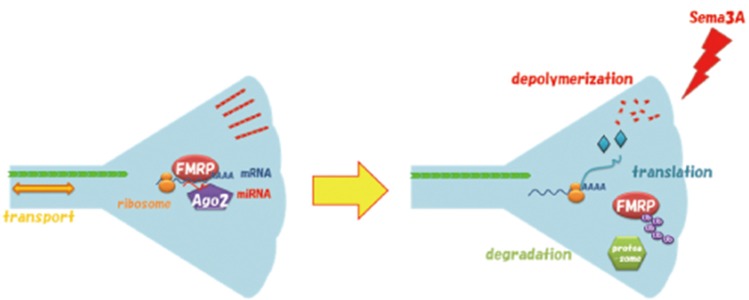
Model for Sema3A-induced degradation of FMRP in growth cones *via* the ubiquitin-proteasome pathway. FMRP interacts with mRNA and Argonaute 2 (Ago2) bound to microRNA (miRNA) in the growth cone before stimulation. Sema3A promotes ubiquitination of FMRP resulting in degradation, which initiates the translation of MAP1B and other cytoskeleton-related proteins for growth cone collapse. Ub, Ubiquitin.

We previously suggested that Sema3A induced growth cone collapse in a translation-dependent fashion (Li et al., 2009). Thus, it is possible that growth cone collapse is also mediated by this pathway *via* local protein synthesis, such as MAP1B. To investigate this possibility, we examined the effect of ubiquitin-proteasome inhibitors on Sema3A-induced growth cone collapse. When Sema3A was applied to hippocampal neurons at 0.1 nM, about 45% of growth cones exhibited a collapsed morphology (Figure 6). MG132 and PYR-42 significantly suppressed the Sema3A-induced collapse compared with controls (Figure 6). Taken together, these results support the involvement of the ubiquitin-proteasome pathway in Sema3A-induced MAP1B translation in growth cones and growth cone collapse (Figure 7).

## Discussion

In this study, we showed that FMRP degradation in growth cones is involved in Sema3A signaling. Localization and traffic of FMRP have been investigated in dendrites and postsynapses, where FMRP reportedly regulates local translation for synaptic plasticity (Huber et al., 2002; Hou et al., 2006). However, FMRP is also localized and trafficked in axons and growth cones (Antar et al., 2006; Hengst et al., 2006; Price et al., 2006; Murashov et al., 2007). Notably, these studies described alterations in axon elongation and projections in FXS model mouse neurons (Antar et al., 2006; Bureau et al., 2008), suggesting that FMRP is also important for axonal functions such as axonal outgrowth and axon guidance. Recently, we reported that FMRP was involved in Sema3A-induced growth cone collapse in a protein-synthesis-dependent manner (Li et al., 2009). However, it remains unclear how FMRP regulated translation of its target mRNAs in response to Sema3A stimulation to elicit morphological changes of growth cones. Here, we demonstrated that FMRP degradation *via* the ubiquitin-proteasome system regulates mRNA translation, resulting in Sema3A-induced growth cone collapse (Figure 7). These findings provided new insight into the mechanisms by which FMRP regulates translation for axon guidance.

We demonstrated that Sema3A induces decreased FMRP levels in growth cones of cultured mouse hippocampal neurons. Decreased FMRP levels were previously observed in synapses (Antar et al., 2004) and dendrites (Nalavadi et al., 2012) after stimulation with group 1 metabotropic glutamate receptors (mGluRs). These decreases in FMRP can be explained by two possibilities: retrograde transport of FMRP mediated by dynein and/or local degradation of FMRP mediated by the ubiquitin-proteasome pathway. Our pharmacological analysis revealed that local degradation *via* the ubiquitin-proteasome pathway, but not retrograde transport *via* dynein, is involved in Sema3A-induced decreases in FMRP levels in growth cones. As mGluR stimulation-induced decreases in FMRP level in dendrites were previously shown to result from degradation *via* the ubiquitin-proteasome pathway (Nalavadi et al., 2012), local degradation of FMRP is considered to be a common mechanism to regulate its function, such as local translation between growth cones of axons and synapses of dendrites. Several groups have reported that ubiquitination in growth cones is regulated by axon guidance factors. For example, nerve growth factor application led to degradation of RhoA mediated by Smurf1, an E3 ubiquitin ligase, in growth cones of cultured rat dorsal root ganglion neurons for axonal growth (Deglincerti et al., 2015). In addition, netrin-1 induced deubiquitination of the actin regulatory protein VASP, which is ubiquitinated by the E3 ubiquitin ligase TRIM9, to regulate filopodia stability of growth cones of mouse cortical neurons (Menon et al., 2015). Although FMRP is reportedly ubiquitinated by Cdh1-APC, an E3 ubiquitin ligase, to mediate mGluR-dependent synaptic plasticity (Huang et al., 2015), it remains unclear which E3 ubiquitin ligase mediates the Sema3A-induced decrease in FMRP in growth cones for the collapse response. Thus, future work is necessary to identify the E3 ubiquitin ligase for ubiquitination and degradation of FMRP after Sema3A stimulation, and to clarify the mechanism by which local translation for growth cone collapse is regulated by FMRP degradation.

Previously, we showed that Sema3A increases MAP1B levels in growth cones and axons in an FMRP- and protein-synthesis-dependent manner, suggesting that Sema3A-induced local translation of MAP1B is mediated by FMRP (Li et al., 2009). In this study, we demonstrated that the Sema3A-induced MAP1B increase in growth cones was suppressed by ubiquitin-proteasome pathway inhibitors. Taken together, our findings suggest that local translation of MAP1B in growth cones was elicited by the degradation of FMRP *via* the ubiquitin-proteasome pathway. MG132 reportedly suppressed the DHPG-induced increase in luciferase activity of a reporter containing the 3′-UTR of PSD-95 (Nalavadi et al., 2012), which is responsible for translational regulation by FMRP. Thus, these findings suggest the possibility that FMRP degradation by the ubiquitin-proteasome pathway in response to activation by extracellular signals promotes the translation of FMRP-target mRNAs, such as MAP1B and PSD-95. However, it is not fully understood how locally translated proteins triggered by FMRP degradation regulate growth cone morphology. As MAP1B regulates the flexibility of microtubules and is an F-actin-binding protein (Cammarata et al., 2016), local increases in MAP1B are considered to induce cytoskeletal reorganization of both microtubules and microfilaments. Another candidate is cofilin 1, an actin-binding protein that regulates the depolymerization of actin filaments (Tilve et al., 2015; Pyronneau et al., 2017; Choi et al., 2018). Interestingly, reduction of cofilin levels in hippocampal neurons attenuated responses to Sema3A-induced growth cone turning (Aizawa et al., 2001). Moreover, cofilin 1 mRNA binds to FMRP, which regulates its translational activity (Feuge et al., 2019). The local supply of these cytoskeletal-related proteins by local translation in growth cones may affect the morphology of growth cones for axon guidance. Thus, future investigation is required to delineate which cytoskeletal-related proteins translated under FMRP control by the ubiquitin-proteasome pathway are involved in morphological changes of growth cones. Although a previous report showed that Sema3A did not increase levels of mono- and polyubiquitinated protein conjugates in growth cones of *Xenopus* retinal neurons (Campbell and Holt, 2001), our results indicate that Sema3A significantly increased ubiquitinated protein conjugates in the growth cones of mouse hippocampal neurons. One possibility for this discrepancy is that dependency of Sema3A signaling on the ubiquitin-proteasome pathway may vary across neuronal types and species. Thus, additional work is needed to identify the ubiquitin-proteasome pathway for FMRP in growth cones.

In conclusion, our findings demonstrate that FMRP degradation in response to Sema3A was mediated by the ubiquitin-proteasome pathway. This work provides new insight into the involvement of FMRP in axon guidance. Future work is necessary to examine whether asymmetrical ubiquitination and degradation of FMRP occur in growth cones in response to a gradient of Sema3A. Our findings have important implications for understanding the formation of neural circuits in the pathophysiological condition of FXS.

## Data Availability Statement

The datasets generated for this study are available on request to the corresponding author.

## Ethics Statement

All animal procedures were performed according to guidelines outlined in the Institutional Animal Care and Use Committee of the Yokohama City University.

## Author Contributions

MT discussed experiments, performed experiments, and wrote the manuscript. YG optimized and performed Sema3A production. YS designed experiments and wrote the manuscript.

## Conflict of Interest

The authors declare that the research was conducted in the absence of any commercial or financial relationships that could be construed as a potential conflict of interest.

## Supplementary Material

VIDEO S1 Retrograde transport visualized by LysoTracker dye in axons of cortical neurons in neuron ball culture. A 1-min video sequence of LysoTracker signal is shown with images captured every 1 s; the illustrated video displays 10 frames/s. Axons of neuron balls extend from left to right in this video. To easily identify axons, a DIC image is presented at the initial 3 s. Note that retrograde transport actively occurred in all axons.

VIDEO S2 Effect of pretreatment with EHNA, a dynein ATPase inhibitor, on retrograde transport in axons of cortical neurons in neuron ball culture. Neuron balls were pretreated with EHNA (50 μM) for 10 min, and then the LysoTracker signal was captured. A 1-min video sequence of LysoTracker signal is shown with images captured every 1 s; the illustrated video displays 10 frames/s. Axons of neuron balls extend from left to right in this video. To easily identify axons, the DIC image is presented at the initial 3 s. Note that retrograde transport was almost stopped in axons in the presence of EHNA.
